# The national burden of influenza‐associated severe acute respiratory illness hospitalization in Rwanda, 2012‐2014

**DOI:** 10.1111/irv.12494

**Published:** 2017-12-02

**Authors:** José Nyamusore, Joseph Rukelibuga, Mwumvaneza Mutagoma, Andrew Muhire, Alice Kabanda, Thelma Williams, Angela Mutoni, Julius Kamwesiga, Thierry Nyatanyi, Jared Omolo, Adeline Kabeja, Jean Baptiste Koama, Agrippine Mukarurangwa, Jeanne d'Arc Umuringa, Carolina Granados, Michel Gasana, Ann Moen, Stefano Tempia

**Affiliations:** ^1^ Epidemic Surveillance and Response Division Rwanda Biomedical Center Ministry of Health Kigali Rwanda; ^2^ Influenza Program Centers for Disease Control and Prevention Kigali Rwanda; ^3^ Health Management Information System Division Ministry of Health Kigali Rwanda; ^4^ National Reference Laboratory Rwanda Biomedical Center Ministry of Health Kigali Rwanda; ^5^ Influenza Division Centers for Disease Control and Prevention Atlanta GA USA; ^6^ Institute of HIV/AIDS Disease Prevention and Control Rwanda Biomedical Center Ministry of Health Kigali Rwanda; ^7^ Influenza Program Centers for Disease Control and Prevention Pretoria South Africa; ^8^ Centre for Respiratory Diseases and Meningitis National Institute for Communicable Diseases of the National Health Laboratory Service Johannesburg South Africa

**Keywords:** burden, hospitalization, influenza, Rwanda, severe acute respiratory illness

## Abstract

**Background:**

Estimates of influenza‐associated hospitalization are severely limited in low‐ and middle‐income countries, especially in Africa.

**Objectives:**

To estimate the national number of influenza‐associated severe acute respiratory illness (SARI) hospitalization in Rwanda.

**Methods:**

We multiplied the influenza virus detection rate from influenza surveillance conducted at 6 sentinel hospitals by the national number of respiratory hospitalization obtained from passive surveillance after adjusting for underreporting and reclassification of any respiratory hospitalizations as SARI during 2012‐2014. The population at risk was obtained from projections of the 2012 census. Bootstrapping was used for the calculation of confidence intervals (CI) to account for the uncertainty associated with all levels of adjustment. Rates were expressed per 100 000 population. A sensitivity analysis using a different estimation approach was also conducted.

**Results:**

SARI cases accounted for 70.6% (9759/13 813) of respiratory admissions at selected hospitals: 77.2% (6783/8786) and 59.2% (2976/5028) among individuals aged <5 and ≥5 years, respectively. Overall, among SARI cases tested, the influenza virus detection rate was 6.3% (190/3022): 5.7% (127/2220) and 7.8% (63/802) among individuals aged <5 and ≥5 years, respectively. The estimated mean annual national number of influenza‐associated SARI hospitalizations was 3663 (95% CI: 2930‐4395—rate: 34.7; 95% CI: 25.4‐47.7): 2637 (95% CI: 2110‐3164—rate: 168.7; 95% CI: 135.0‐202.4) among children aged <5 years and 1026 (95% CI: 821‐1231—rate: 11.3; 95% CI: 9.0‐13.6) among individuals aged ≥5 years. The estimates obtained from both approaches were not statistically different (overlapping CIs).

**Conclusions:**

The burden of influenza‐associated SARI hospitalizations was substantial and was highest among children aged <5 years.

## 1 INTRODUCTION

Influenza virus infections cause substantial morbidity and mortality globally, in particular among individuals aged <5 and ≥65 years and persons with underlying medical conditions.^1^, ^2^, ^3^, ^4^, ^5^, ^6^ In addition, a higher burden of influenza‐associated hospitalization has been reported among African children compared to other regions.^2^, ^3^

Influenza sentinel surveillance among patients hospitalized with severe acute respiratory illness (SARI) has been established in several African countries in the past decade,^7^ and such data have contributed to global studies on influenza‐associated hospitalization.^2^, ^3^ Nonetheless, such studies were limited to a pediatric population. Estimates of the national burden of influenza‐associated hospitalization across age groups are severely limited in Africa, having been described only in 3 countries on the continent.^8^, ^9^, ^10^

Although the burden of influenza‐associated severe illness may be higher in Africa compared to other regions,^2^, ^3^ the use of influenza vaccine and antivirals on the continent, including Rwanda, remains limited.^11^ The World Health Organization (WHO) under the Global Influenza Program highlighted that there is a need for influenza disease burden estimates especially from low‐ and middle‐income countries.^12^ These estimates would enable governments to make informed evidence‐based decisions when allocating scarce resources and planning intervention strategies to limit the impact and spread of the disease. In addition, national estimates would contribute to the global understanding of the burden of influenza‐associated severe illness and guide policies at national and global levels.

We aimed to estimate the national and provincial number and rates of SARI and influenza‐associated SARI hospitalizations among different age groups in Rwanda from January 2012 through December 2014.

## 2 METHODS

### 2.1 Data sources

#### 2.1.1 Data source 1: National number of respiratory hospitalizations

We obtained national numbers of respiratory hospitalizations from the Rwanda Health Management Information System (HMIS), which collects aggregated and de‐identified data on number of admission by syndrome from all public hospitals in Rwanda^13^, ^14^ from January 2012 through December 2014.

#### 2.1.2 Data source 2: Retrospective record review of respiratory admissions in selected hospitals

To assess the completeness of data reported through the HMIS and estimate the proportion of SARI cases among any respiratory admission, we implemented an anonymized retrospective record review (using hospital admission books) of any respiratory admission in 6 (13%) of 45 public hospitals conducting influenza sentinel surveillance situated in each of the 5 provinces of the country over the same study period. Consistent with HMIS reporting practices, respiratory admissions were considered patients admitted with a clinician diagnosis of lower respiratory tract infection, pneumonia, bronchopneumonia, bronchitis, or any other pneumopathy. For each identified respiratory admission, gender, age, presence of fever or history of fever and cough, duration of symptoms, and date and place of admission were recorded.

#### 2.1.3 Data source 3: Influenza virus surveillance among patients hospitalized with severe acute respiratory illness

We conducted active prospective hospital‐based surveillance for SARI at 6 public hospitals located in each of the 5 provinces of Rwanda (the Kigali University Teaching Hospital and the Kibagabaga District Hospital in Kigali City, the Butare University Teaching Hospital in Southern Province, the Kibungo District Hospital in Eastern Province, the Gihundwe District Hospital in Western Province, and the Ruhengeri District Hospital in Northern Province) from January 2012 through December 2014. Case detection was conducted at the pediatric, adult, and maternity inpatient wards of each hospital.

A case of SARI was defined as a hospitalized person of any age presenting with either temperature ≥38°C or history of fever and cough of duration of ≤10 days.^15^

The procedures of this surveillance program have been previously described.^16^ Briefly, surveillance nurses completed case report forms that included demographic, clinical and epidemiological information for all enrolled SARI cases. In addition, respiratory specimens (nasopharyngeal and oropharyngeal swabs) were collected from all enrolled patients, placed in universal transport medium, stored at 4‐8°C and transported to the National Reference Laboratory in Kigali within 72 hours of collection for testing. Specimens were tested for influenza type A and B viruses using a real‐time reverse transcriptase polymerase chain reaction assay.^16^ Influenza A‐positive samples were further subtyped.^17^

#### 2.1.4 Data source 4: Population denominators

National and provincial age‐ and year‐specific population denominators were obtained from projections of the 2012 Rwanda census data.^18^ Rwanda had a population of 10 955 840 individuals in 2014 of which 1 604 444 (14.6%) were children aged <5 years.^18^

### 2.2 Estimation of the number and rate of SARI and influenza‐associated SARI hospitalizations

To estimate the national number of SARI and influenza‐associated SARI hospitalization, we used 2 different methods.

#### 2.2.1 Method 1

In Method 1, we used a four‐step approach.

In Step 1.1, we estimated the national number of any respiratory hospitalization by multiplying the national number of respiratory hospitalization reported through the HMIS (data source 1) by the estimated proportion of underreporting. The proportion of underreporting was obtained by comparing the number of respiratory admissions reported through the HMIS (data source 1) from the hospitals where the retrospective record review was implemented and those recorded from the record review (data source 2).

In Step 1.2, we estimated the national number of SARI cases by multiplying the adjusted number of respiratory hospitalizations obtained in Step 1.1 by the estimated proportion of SARI cases among any respiratory hospitalization. To obtain the proportion of SARI cases among any respiratory hospitalization, we first reclassified any respiratory admission obtained from the record review as SARI if they met the SARI case definition using the clinical information collected during the review (data source 2). Subsequently, we calculated the estimated proportion of SARI cases among any respiratory hospitalization by dividing the number of reclassified SARI cases by the total number of recorded respiratory admission (SARI and non‐SARI).

In Step 1.3, we estimated the national number of influenza‐associated SARI hospitalization by multiplying the number of SARI cases obtained in Step 2 by the influenza virus detected rate obtained from influenza sentinel surveillance implemented among inpatients with SARI (data source 3). For this estimation, we used annual influenza virus detection rate because the number of SARI cases enrolled did not display seasonal variations (data not shown).

In Step 1.4, we estimated the rates of SARI and influenza‐associated SARI hospitalizations by dividing the estimated corresponding numbers obtained in Step 1.3 by the population at risk (data source 4).

The following equations were used to obtain the rates of SARI and influenza‐associated SARI for Method 1:

(1) $${\text{Rate}}_{\mathrm{SARI}}=\frac{\frac{\text{HMIS}}{{\text{Adj}}_{\mathrm{PropRep}}}*{\text{Prop}}_{\mathrm{SARI}}}{\text{Pop}}*100\;000$$

(2) $${\text{Rate}}_{\mathrm{Inf}}={\text{Rate}}_{\mathrm{SARI}}*{\text{Prop}}_{\mathrm{Inf}}$$

where Rate_SARI_ is the rate of SARI hospitalization; HMIS is the national number of respiratory hospitalization reported through the HMIS (data source 1); Adj_PropRep_ is the proportion of respiratory admissions reported through the HMIS (data source 1) from the hospitals where the retrospective record review was implemented and those recorded from the record review (data source 2) obtained in Step 1.1; Prop_SARI_ is the proportion of any respiratory hospitalization obtained from the record review that met the SARI cases definition (data source 2) obtained in Step 1.2; Pop is the population at risk (data source 4); Rate_Inf_ is the rate of influenza‐associated SARI hospitalization; and Prop_Inf_ is the detection rate of influenza virus among SARI cases tested (data source 3).

#### 2.2.2 Method 2

In Method 2, we used a three‐step approach.

In Step 2.1, we estimated the SARI hospitalization rates at the hospitals where influenza sentinel surveillance was conducted using the WHO guidelines for estimating the disease burden associated with seasonal influenza.^12^ First, we estimated the service population of the 6 sentinel sites by dividing the number of HMIS respiratory hospitalizations at each of the sentinel sites by the national number of respiratory hospitalizations reported through the HMIS (data source 1). This proportion was then applied to the population at risk over the study period (data source 4). Thereafter, we obtained the SARI hospitalization rates for the sentinel sites by dividing the total number of SARI hospitalizations at the sentinel sites (data source 2) by the estimated service population.

In Step 2.2, we estimated the rates of influenza‐associated SARI hospitalization at the sentinel sites by multiplying the rates of SARI hospitalizations obtained in Step 2.1 by the influenza virus detected rate obtained from influenza sentinel surveillance implemented among inpatients with SARI (data source 3). We used the SARI and influenza‐associated SARI hospitalization rates at the sentinel sites as a proxy for the corresponding province as previously described.^8^, ^9^

In Step 2.3, we estimated the number of SARI and influenza‐associated SARI hospitalization by multiplying the corresponding rates (obtained in Step 2.2) by the population at risk.

The following equations were used to obtain the rates of SARI and influenza‐associated SARI for Method 2:

(3) $${\text{Rate}}_{\mathrm{SARI}}=\frac{\text{SARI}}{\text{Pop}}*100\;000$$

(4) $${\text{Rate}}_{\mathrm{Inf}}={\text{Rate}}_{\mathrm{SARI}}*{\text{Prop}}_{\mathrm{Inf}}$$

where Rate_SARI_ is the rate of SARI hospitalization; SARI is the number of SARI cases obtained from the record review (data source 2); Pop is the population at risk obtained in Step 2.1; Rate_Inf_ is the rate of influenza‐associated SARI hospitalization; and Prop_Inf_ is the detection rate of influenza virus among SARI cases tested (data source 3).

All analyses for Methods 1 and 2 were implemented overall and within the following age categories: <1, 1‐4, 5‐24, 25‐44, 45‐64, ≥65, <5, and ≥5 years of age. Provincial estimates were also calculated. Rates were expressed per 100 000 population. All estimates were reported as mean annual estimates over the study period.

We obtained the 95% confidence intervals (CI) using bootstrap resampling over 1000 replications for all relevant parameters included in the calculations for each method. This included (i) the age‐, year‐, and hospital‐specific proportion of underreporting (including interhospital variability), (ii) the age‐ and year‐specific proportion of SARI cases over total respiratory admissions, and (iii) the age‐ and year‐specific influenza virus detection rate among SARI cases tested. The lower and upper limits of the 95% CI were the 2.5th and 97.5th percentiles of the estimated values obtained from the 1000 resampled datasets, respectively.

The statistical analysis was implemented using Stata 14.1 (StataCorp, College Station, TX, USA).

### 2.3 Ethics

The influenza sentinel surveillance and the retrospective record review were deemed non‐research by the Rwanda Ministry of Health and the US Centers for Disease Control and Prevention. The HMIS and census data were publicly available.

## 3 RESULTS

### 3.1 Number of respiratory hospitalizations and retrospective record review of respiratory admissions in selected hospitals

During 2012‐2014, there were 13 813 respiratory admissions recorded from the retrospective record review at the 6 selected hospitals, of which 10 801 (78.2%) were reported through the HMIS: 7169/8786 (81.6%) and 3632/5028 (72.2%) among individuals aged <5 and ≥5 years, respectively. At the hospitals selected for the record review, SARI cases accounted for 70.6% (9759/13 813) of respiratory admissions: 77.2% (6783/8786) and 59.2% (2976/5028) among individuals aged <5 and ≥5 years, respectively. Children aged <5 years accounted for 80.0% (6783/9759) of total SARI hospitalizations.

### 3.2 Influenza virus surveillance among patients hospitalized with SARI

**Figure 1:**
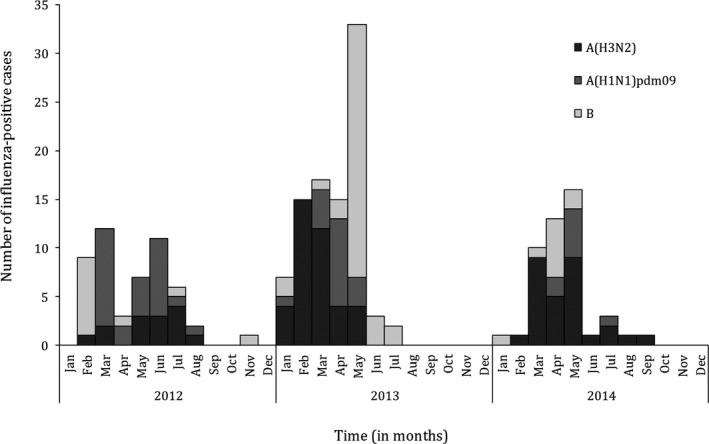
Monthly number of influenza‐positive cases among patients hospitalized with severe acute respiratory illness at 6 sentinel sites, Rwanda, 2012‐2014

During the study period, we enrolled and tested 3022 SARI cases: 2220 (73.5%) and 802 (26.5%) cases among individuals aged <5 and ≥5 years, respectively. Influenza viruses were detected in 190 (6.3%) specimens: 127 (5.7%) and 63 (7.8%) among individuals aged <5 and ≥5 years, respectively. Of these, 81 (42.6%) were influenza A(H3N2), 52 (27.3%) were influenza A(H1N1)pdm09, and 57 (30.0%) were influenza B. Influenza viruses were detected mainly from January to July, the long rainy season in Rwanda (Figure 1).

### 3.3 National number and rate of SARI and influenza‐associated SARI hospitalizations

Using Method 1, the estimated mean annual number of SARI hospitalization was 60 957 (95% CI: 48 766‐73 148) (rate: 571.1; 95% CI: 456.9‐685.3 per 100 000 population): 48 043 (95% CI: 38 434‐57 652) (78.8%) (rate: 3073.6; 95% CI: 2485.9‐3688.3 per 100 000 population) and 12 915 (95% CI: 10 332‐15 495) (21.2%) (rate: 141.8; 95% CI: 113.4‐170.2 per 100 000 population) among individuals aged <5 and ≥5 years, respectively (Table 1). The estimated mean annual rates of SARI hospitalization was highest among children aged <1 year (6912.8; 95% CI: 5530.2‐8295 per 100 000 population) and lowest among individuals aged 5‐24 years (116.4; 95% CI: 93.1‐135.7 per 100 000 population).

**Table 1:** Estimated mean annual numbers and rates of severe acute respiratory illness and influenza‐associated severe acute respiratory illness hospitalizations, Rwanda, 2012‐2014 (Method 1)

Age group (in years)	SARI hospitalizations		Influenza‐associated SARI hospitalizations	
	Number (95% CI)	Rate (95% CI)^a^	Number (95% CI)	Rate (95% CI)^a^
All	60 957 (48 766‐73 148)	571.1 (456.9‐685.3)	3663 (2930‐4396)	34.7 (25.4‐47.7)
<1	21 720 (17 376‐26 064)	6912.8 (5530.2‐8295.4)	927 (742‐1112)	295.0 (236.2‐354.7)
1‐4	26 323 (21 058‐31 588)	2107.7 (1686.2‐2529.2)	1710 (1368‐2052)	136.9 (109.5‐164.3)
5‐24	5825 (4660‐6990)	116.4 (93.1‐135.7)	546 (437‐655)	10.9 (8.7‐13.1)
25‐44	3414 (2731‐4097)	128.7 (103.0‐154.4)	227 (182‐272)	8.6 (6.9‐10.3)
45‐64	2306 (1845‐2767)	207.1 (165.7‐248.5)	136 (109‐163)	12.2 (9.8‐14.6)
≥65	1370 (1096‐1644)	402.5 (322.0‐483.0)	117 (94‐140)	34.3 (27.4‐41.2)
Age group (in 2 categories)				
<5	48 043 (38 434‐57 652)	3073.6 (2458.9‐3688.3)	2637 (2110‐3164)	168.7 (135.0‐202.4)
≥5	12 915 (10 332‐15 495)	141.8 (113.4‐170.2)	1026 (821‐1231)	11.3 (9.0‐13.6)
Province				
Kigali City	6100 (4880‐7320)	518.3 (414.6‐662.0)	366 (293‐439)	28.3 (22.6‐34.0)
Eastern	12 505 (10 004‐15 006)	470.1 (376.1‐564.1)	752 (602‐902)	31.1 (24.9‐37.3)
Western	15 958 (12 766‐19 150)	640.5 (512.4‐768.6)	959 (767‐1151)	43.5 (34.8‐52.2)
Northern	12 543 (10 034‐15 052)	724.1 (579.3‐868.9)	754 (603‐905)	31.9 (25.5‐38.3)
Southern	13 851 (11 081‐16 621)	530.1 (424.1‐636.1)	832 (666‐998)	38.5 (30.8‐46.2)

SARI, severe acute respiratory illness; CI, confidence intervals.

a Rates expressed per 100 000 population.

Using Method 1, the estimated mean annual number of influenza‐associated SARI hospitalization was 3663 (95% CI: 2930‐4395) (rate: 34.3; 95% CI: 25.4‐47.7 per 100 000 population): 2637 (95% CI: 2110‐3164) (72.0%) (rate: 168.7; 95% CI: 135.0‐202.4 per 100 000 population) and 1026 (95% CI: 821‐1231) (28.0%) (rate: 11.3; 95% CI: 9.0‐13.6 per 100 000 population) among individuals aged <5 and ≥5 years, respectively (Table 1). The estimated mean annual rates of influenza‐associated SARI hospitalization were highest among children aged <1 year (295.0; 95% CI: 236.2‐354.7 per 100 000 population) and lowest among individuals aged 25‐44 years (8.6; 95% CI: 6.9‐10.3 per 100 000 population).

Using Method 2, the estimated mean annual number of SARI hospitalization was 60 214 (95% CI: 48 539‐73 112): 47 923 (95% CI: 36 823‐56 923) among children aged <5 years and 12 291 (95% CI: 9974‐15 837) among individuals aged ≥5 years (Table 2).

**Table 2:** Estimated mean annual numbers and rates of severe acute respiratory illness and influenza‐associated severe acute respiratory illness hospitalizations, Rwanda, 2012‐2014 (Method 2)

Age group (in years)	SARI hospitalizations		Influenza‐associated SARI hospitalizations	
	Number (95% CI)	Rate (95% CI)^a^	Number (95% CI)	Rate (95% CI)^a^
All	60 214 (48 539‐73 112)	568.8 (414.9‐721.7)	3648 (2663‐4633)	34.2 (25.0‐43.4)
<1	19 178 (15 655‐22 435)	6104.0 (5134‐7025)	819 (655‐982)	260.5 (208.4‐312.6)
1‐4	28 745 (23 816‐33 367)	2301.6 (1798.2‐2923.6)	1867 (1438‐2297)	149.5 (115.1‐183.9)
5‐24	5039 (4021‐6827)	100.7 (70.5‐130.9)	472 (331‐614)	9.4 (6.6‐12.3)
25‐44	3001 (2498‐3627)	113.1 (82.6‐143.7)	200 (146‐253)	7.6 (5.5‐9.6)
45‐64	2736 (2023‐3071)	245.8 (169.6‐322.0)	161 (111‐211)	14.5 (10.0‐19.0)
≥65	1514 (1145‐1934)	444.8 (315.8‐573.7)	129 (92‐167)	37.9 (26.9‐48.9)
Age group (in 2 categories)				
<5	47 923 (36 823‐56 923)	3066.0 (2391.4‐3740.5)	2686 (2095‐3277)	171.8 (134.0‐209.6)
≥5	12 291 (9974‐15 837)	139.8 (99.3‐180.3)	962 (683‐1242)	10.6 (7.5‐13.6)
Province				
Kigali City	6623 (4968‐8279)	562.8 (422.1‐703.5)	401 (301‐502)	34.1 (25.6‐42.6)
Eastern	11 441 (8123‐14 758)	430.1 (305.4‐554.8)	693 (492‐894)	26.1 (18.5‐33.6)
Western	15 054 (10 387‐19 720)	604.2 (416.9‐791.5)	912 (629‐1195)	36.6 (25.3‐48.0)
Northern	13 849 (10 802‐16 896)	799.5 (623.6‐975.4)	839 (655‐1024)	48.4 (37.8‐59.1)
Southern	13 247 (9803‐16 691)	507.0 (375.2‐638.8)	803 (594‐1011)	30.7 (22.7‐38.5)

SARI, severe acute respiratory illness; CI, confidence intervals.

a Rates expressed per 100 000 population.

Using Method 2, the estimated mean annual number of influenza‐associated SARI hospitalization was 3648 (95% CI: 2663‐4633): 2686 (95% CI: 2095‐3277) among children aged <5 years and 962 (95% CI: 683‐1242) among individuals aged ≥5 years (Table 2).

The estimated number and rates of SARI and influenza‐associated SARI hospitalizations were similar (overlapping CIs) between the 2 estimation approaches (Tables 1 and 2).

**Figure 2:**
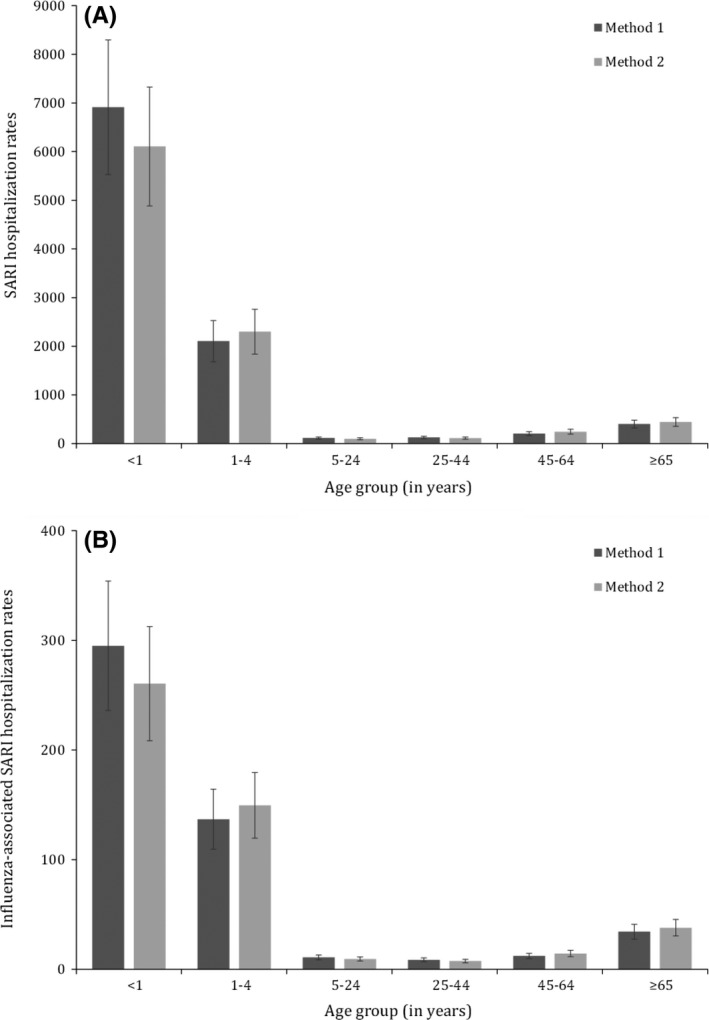
Mean annual estimates of severe acute respiratory illness (SARI) and influenza‐associated severe acute respiratory illness hospitalization rates (per 100 000 population) by age group, Rwanda, 2012‐2014. (A) SARI hospitalization rates; (B) influenza‐associated SARI hospitalization rates

A U‐shaped trend of the magnitude of the SARI and influenza‐associated SARI hospitalizations rates was observed across age groups (Tables 1 and 2 and Figure 2). No statistically significant difference (overlapping CIs) of the SARI and influenza‐associated SARI hospitalization rates was observed across Provinces (Tables 1 and 2).

## 4 DISCUSSION

We reported national and provincial estimates of influenza‐associated SARI hospitalization in Rwanda over a 3‐year period. Influenza‐associated SARI hospitalizations were substantial and were observed across age groups. However, the influenza‐associated SARI hospitalization rates were highest among individuals aged <5 and ≥65 years. Children aged <5 years accounted for 72% of the total influenza‐associated SARI hospitalizations.

Higher rates of influenza‐associated respiratory hospitalizations among young children and the elderly have been reported in other case‐based and ecological studies.^4^, ^19^ The estimated rates of influenza‐associated SARI hospitalization among Rwandan children aged <5 years (168.9‐171.8 per 100 000 population) were similar to those reported in South Africa (range: 153‐186 per 100 000 population),^4^ Kenya (270 per 100 000 population),^20^ and Ghana (135 per 100 000 population)^10^ from population‐based studies and global estimates for Africa (174 per 100 000 population),^3^ and such estimates were higher compared to those of other regions.^3^

Among individuals aged ≥5 years, the Rwanda estimates (10.6‐11.3 per 100 000 population) were on the lower level, but overall similar when compared to other estimates from Africa: South Africa (22.1 per 100 000 population)^4^ and Kenya (30.0 per 100 000 population).^20^ Lower estimates in this age group may be due to different prevalence of chronic underlying medical conditions (known risk factors for influenza‐associated severe illness^21^) in different populations. Specifically, HIV infection is a well‐documented risk factor for influenza‐associated severe illness,^4^, ^5^, ^6^, ^21^ and this may have played a role in the observed “higher” rates especially in South Africa where the HIV prevalence in the adult population is elevated.^4^

Similar to South African and Kenyan studies,^8^, ^9^ no significant difference in the provincial rates of influenza‐associated SARI hospitalizations was observed, suggesting that geographical variations within countries may not affect significantly the burden associated with influenza virus infection.

The estimated rates of SARI hospitalization among individuals aged <5 years (3066.0‐3073.6 per 100 000 population) and ≥5 years (139.8‐141.8 per 100 000 population) in Rwanda were also similar to those reported in South Africa from population‐based studies (range: 2530‐3173 per 100 000 population among children aged <5 years^22^ vs 325‐389 per 100 000 population among individuals aged ≥5 years^23^).

In our study, of the patients hospitalized with any respiratory illness at the hospitals where the record review was conducted, 77.2% and 59.2% met the SARI case definition among individuals aged <5 and ≥5 years, respectively. In a South African study where patients hospitalized for respiratory illness of any duration were enrolled, 92% and 56% met the SARI case definition among individuals aged <5 and ≥5 years, respectively.^24^

Our study has limitations that warrant discussion. First, like in most of this kind of studies, we used several adjustment factors to obtain national estimates of influenza‐associated SARI hospitalizations. The accuracy of these adjustment factors may have affected the accuracy of the final national estimates. However, we accounted for the uncertainty of all adjustment factors using bootstrap resampling for the calculation of the CIs. Second, we estimated the burden of influenza‐associated hospitalization only among patients hospitalized with SARI. Influenza virus infection has been reported also among patients hospitalized for respiratory illness that do not meet the SARI case definition. Specifically in a study conducted in South Africa, influenza virus was detected in 5.8% of patients hospitalized with respiratory illnesses that did not meet the SARI case definition.^24^ In addition, ecological studies have suggested that influenza virus is responsible for hospitalizations and deaths also among patients presenting with circulatory illnesses or even non‐respiratory and non‐circulatory syndromes.^5^, ^6^, ^19^ Third, we did not collect data on the total number of medical hospitalizations, hindering our ability to estimate the proportional contribution of SARI‐associated admissions among any medical admission. Lastly, individuals that may have developed influenza‐associated severe illness, but did not seek care would have been missed in our study; hence, our estimates should be considered minimum estimates.

In conclusion, we reported a substantial hospitalization burden associated with influenza virus infection especially in individuals aged <5 and ≥65 years. Our estimates were similar to those from other countries from Africa or global estimates for Africa but higher than those estimated from other regions, highlighting the heavier burden of influenza‐associated severe illness on the continent. The RMoH is yet to implement a national influenza vaccination program. Should an influenza vaccination program be introduced in Rwanda, young children and the elderly may benefit most from annual influenza immunization. No influenza vaccine is licensed for children aged <6 months, but this group may be protected through the vaccination of their mothers during pregnancy.^25^, ^26^

## DISCLAIMER

The findings and conclusions in this report are those of the authors and do not necessarily represent the official position of the US Centers for Disease Control and Prevention, USA, or the Rwanda Ministry of Health.

## CONFLICT OF INTEREST

All authors declare that they have no commercial or other associations that may pose a conflict of interest.

## AUTHOR CONTRIBUTIONS

All authors take responsibility for the integrity of the data and the accuracy of the data analysis. Stefano Tempia, José Nyamusore, Jared Omolo, Jean Baptiste Koama, and Joseph Rukelibuga involved in study concept and design. José Nyamusore, Joseph Rukelibuga, Jared Omolo, Stefano Tempia, Mwumvaneza Mutagoma, Angela Mutoni, Andrew Muhire, Julius Kamwesiga, Adeline Kabeja, Alice Kabanda, Jeanne d'Arc Umuringa, and Agrippine Mukarurangwa involved in acquisition, analysis, or interpretation of data. José Nyamusore and Stefano Tempia drafted the manuscript. Thelma Williams, Carolina Granados, Ann Moen, Michel Gasana, Joseph Rukelibuga, Stefano Tempia, and Mwumvaneza Mutagoma critically revised the manuscript for important intellectual content.
